# Comparison of Postural Responses to Galvanic Vestibular Stimulation between Pilots and the General Populace

**DOI:** 10.1155/2015/567690

**Published:** 2015-01-06

**Authors:** Yang Yang, Fang Pu, Xiaoning Lv, Shuyu Li, Jing Li, Deyu Li, Minggao Li, Yubo Fan

**Affiliations:** ^1^International Joint Research Center of Aerospace Biotechnology and Medical Engineering of Ministry of Science and Technology of China, School of Biological Science and Medical Engineering, Beihang University, No. 37 Xueyuan Road, Haidian District, Beijing 100191, China; ^2^State Key Laboratory of Virtual Reality Technology and Systems, Beihang University, No. 37 Xueyuan Road, Haidian District, Beijing 100191, China; ^3^Aviation and Nautical Medical Center, Navy General Hospital, No. 6 Fucheng Road, Haidian District, Beijing 100048, China

## Abstract

Galvanic vestibular stimulation (GVS) can be used to study the body's response to vestibular stimuli. This study aimed to investigate whether postural responses to GVS were different between pilots and the general populace. Bilateral bipolar GVS was applied with a constant-current profile to 12 pilots and 12 control subjects via two electrodes placed over the mastoid processes. Both GVS threshold and the center of pressure's trajectory (COP's trajectory) were measured. Position variability of COP during spontaneous body sway and peak displacement of COP during GVS-induced body sway were calculated in the medial-lateral direction. Spontaneous body sway was slight for all subjects, and there was no significant difference in the value of COP position variability between the pilots and controls. Both the GVS threshold and magnitude of GVS-induced body deviation were similar for different GVS polarities. GVS thresholds were similar between the two groups, but the magnitude of GVS-induced body deviation in the controls was significantly larger than that in the pilots. The pilots showed less GVS-induced body deviation, meaning that pilots may have a stronger ability to suppress vestibular illusions.

## 1. Introduction

The primary function of the vestibular system is to detect motion and head position. Specifically, three semicircular canals can perceive angular acceleration and velocity of the head, and the otolith organs (utricle and saccule) can sense linear acceleration of the head and head tilt [1–3]. During flight, especially during high levels of translational acceleration and/or angular acceleration, pilots may respond abnormally to vestibular stimuli and suffer disequilibrium, vertigo, spatial disorientation (SD), and motion sickness, which may have serious safety implications [4–6]. Therefore, the ability to respond to vestibular stimuli is important for pilot selection and training.

Galvanic vestibular stimulation (GVS) is a technique that can stimulate the vestibular nerves associated with both semicircular canals and otolith organs [1, 7]. By applying a small current through a surface electrode over the mastoid process behind the ear, the firing rate of all vestibular afferents can be changed. These changes are the same as those arising from rotating the head [8, 9]. Therefore, the central nervous system (CNS) could mistakenly consider GVS as a head movement and then the whole-body responses can be evoked [1]. Using different configurations of GVS will generate different perceived directions of illusory head movements [10–12]. In detail, bilateral bipolar GVS with an anodal electrode placed over one mastoid process and a cathodal electrode placed over the other mastoid process will produce a roll like sensation [1]. In addition, these illusory movements evoked by GVS are similar to the leans, which is the most common vestibular illusion in flight. GVS can provide a safe and convenient way to simulate SD [13, 14].

Because of its simple and harmless nature, GVS has been widely adopted as a research tool for probing the body's responses to vestibular stimuli [15–17], such as eye movement [18, 19], standing balance [7, 20, 21], walking [22, 23], and hand movements [14, 24]. The direction of body sway evoked by bilateral bipolar GVS is always towards the anodal ear along the interaural line [21]. Furthermore, GVS-induced deviation in both walking [22, 23] and hand movement [14, 24] is also towards the anode side. However, some studies have discovered that the amplitude of sway varies from person to person [25–28]. Balter et al. observed that GVS-induced body sway in adult women was significantly greater than that in both gymnasts and untrained adolescents [25], but the differences in GVS-induced body sway between carsick and healthy subjects were not significant [26]. Rinalduzzi et al. discovered that patients with polyneuropathy had significantly greater postural responses to GVS in comparison with healthy subjects, which suggested that GVS can be useful in detecting people with different vestibular functions [27]. Tax et al. found similar results, reporting that the magnitude of body deviation induced by GVS for patients with bilateral vestibular failure was significantly smaller than that of healthy people, and concluded that GVS is a viable noninvasive method for assessing vestibular function [28].

Considering that, through pilot selection and training, qualified pilots can bear large vestibular stimuli, the vestibular system of pilots in general may have a different sensitivity and/or different response magnitude to vestibular stimuli than the general populace. This study aimed to investigate whether the postural responses to GVS were different between pilots and the general populace.

## 2. Methods

### 2.1. Subjects

12 pilots (mean age 25.3 yrs, range 24–26 yrs) with 320–400 hours of flying experience and 12 graduate students (mean age 23.8 yrs, range 23–25 yrs) representing the general populace as control subjects participated in the study. None had a history of postural or vestibular deficits. All subjects provided informed consent after being briefed about the experiment, which was approved by the Human Research Ethics Committee of Beihang University.

### 2.2. Equipment

#### 2.2.1. Galvanic Vestibular Stimulation

Bilateral bipolar GVS was achieved using a homemade constant-current generator which was validated and used in our previous study [14]. Constant-current GVS was applied to the subjects via two 2 cm^2^ silver chloride electrodes placed bilaterally over the mastoid processes behind the ears. The resistance between the two electrodes was maintained between 2 and 5 kΩ. The intensity of the GVS current could be varied from 0.0 mA to 1.2 mA in steps of 0.1 mA. The polarity of GVS could be changed by an electronic switch set on the stimulator.

#### 2.2.2. Footscan 0.5 m Plate (RSscan, Olen, Belgium)

A footscan 0.5 m plate (0.5 m × 0.4 m, with 4,096 resistance sensitive sensors, 4 sensors/cm^2^) was used to measure the plantar pressure at a sampling frequency of 100 Hz. Using the footscan 7 gait 2nd generation software, the center of pressure (COP) of the two feet can be exported. And then the COP data were low-pass filtered. Position variability (root mean square of displacement, abbreviated as RMS) of COP during spontaneous body sway and peak displacement of COP during GVS-induced body sway were calculated to quantitatively evaluate the spontaneous body sway and GVS-induced body sway, respectively.

### 2.3. Procedure

#### 2.3.1. GVS Threshold Test

GVS threshold was measured using the stair-case method described by Bent et al. [22] and Wilkinson et al. [29, 30]. The subjects were instructed to naturally stand barefoot with eyes closed, head facing forward, hands hanging by the sides, and feet at an angle of about 30°, according to a study by Cauquil et al. [31]. During the test, subjects were instructed to be relaxed and not to actively oppose the body sway induced by GVS. The electrical current was started at 0.0 mA and increased by 0.1 mA until the threshold was reached, and constant-current GVS lasted for 3 s for each stimulus. The threshold was determined based on the definitive visible body sway judged by the experimenter, in conjunction with the subjective feeling of disorientation [22, 32]. The threshold was then confirmed by reducing the intensity of GVS current by 0.3 mA (if the threshold was less than 0.3 mA, the intensity of GVS current was reduced to 0.0 mA) and then increasing it in 0.1 mA intervals until detectable body sway appeared again. For each subject, GVS thresholds were determined for the anode on the right and the left sides.

#### 2.3.2. Spontaneous and GVS-Induced Body Sway Test

Subjects were asked to stand barefoot on the footscan 0.5 m plate in a relaxed posture similar to the standing posture in the GVS threshold test. Each trial lasted for 7 s, including a 2 s prestimulus period and a 5 s GVS stimulus period. Suprathreshold level (1.0 mA constant-current) GVS was applied. Two kinds of GVS polarities (the anode on the right side and the anode on the left side) were randomly applied. Three trials were performed for each of the two GVS conditions. At the beginning of each trial, the subjects did not know which GVS condition would be presented. A 2 min rest was given between each trial.

### 2.4. Data Analysis

Considering that bilateral bipolar GVS induced the body sway mainly in the medial-lateral direction along the interaural line when the head was facing forward, only the displacement of COP in the medial-lateral direction was analyzed.

COP data exported from footscan 7 gait 2nd generation software were first filtered through a 4th order zero-phase low-pass Butterworth filter with a cutoff frequency of 5 Hz [33].

The amplitude of spontaneous postural sway was quantified by calculating the RMS of the COP in the medial-lateral (*x*) direction (RMS_*x*_) over the 2 s prestimulus period. This parameter is known to be a reliable and valid parameter to evaluate the standing balance and postural sway [33–35]. A greater value of RMS_*x*_ corresponded to lower stability.

The peak postural response to GVS was defined as the difference between the peak COP position in the medial-lateral direction during the 5 s GVS stimulus period and the average COP position calculated over the 2 s prestimulus period in this study, which is commonly used to assess the magnitude of postural responses to GVS [27, 28, 31]. It was calculated by subtracting the average value during the prestimulus period from the peak value during the stimulus period. Negative values signified body deviation to the left from the initial position, whereas positive values signified deviation to the right. Absolute values indicate the magnitude of GVS-induced body deviation, of which greater values mean larger body deviation from the initial position.

### 2.5. Statistical Analysis

All parameters for each trial and in each condition were calculated and then averaged and were expressed as mean ± standard deviation. A one-way ANOVA (2 subject groups) was performed to evaluate the effects of the subject groups on the amplitude of spontaneous body sway during the prestimulus period, and two two-way ANOVAs (2 subject groups × 2 GVS polarities) were used to evaluate the effects of both subject groups and GVS polarities on the GVS thresholds and the magnitude of GVS-induced body deviation.

Statistical tests were performed using the statistical program SPSS 20.0. All significance levels were set at 0.05.

## 3. Results

### 3.1. GVS Thresholds

GVS thresholds of all subjects during quiet stance ranged from 0.1 to 0.6 mA (pilots: 0.1~0.6 mA; control subjects: 0.2~0.6 mA, similar to previous studies [22, 32]). The GVS thresholds of 6 pilots and 5 control subjects were asymmetrical for the anode on the right and the left mastoid processes, and GVS thresholds of the other subjects were symmetrical. The asymmetries between the right and the left thresholds were 0.1 or 0.2 mA. These asymmetries were in accordance with Bent et al. study [22].  Table 1 shows the averaged GVS thresholds of pilots and controls during quiet stance according to different GVS conditions.

The two-way ANOVA revealed that GVS thresholds were not significantly different between pilots and controls [*F*(1,44) = 0.096, *P* = 0.759] and between different GVS polarities [*F*(1,44) = 0.011, *P* = 0.918].

### 3.2. Spontaneous Body Sway

**Figure 1:**
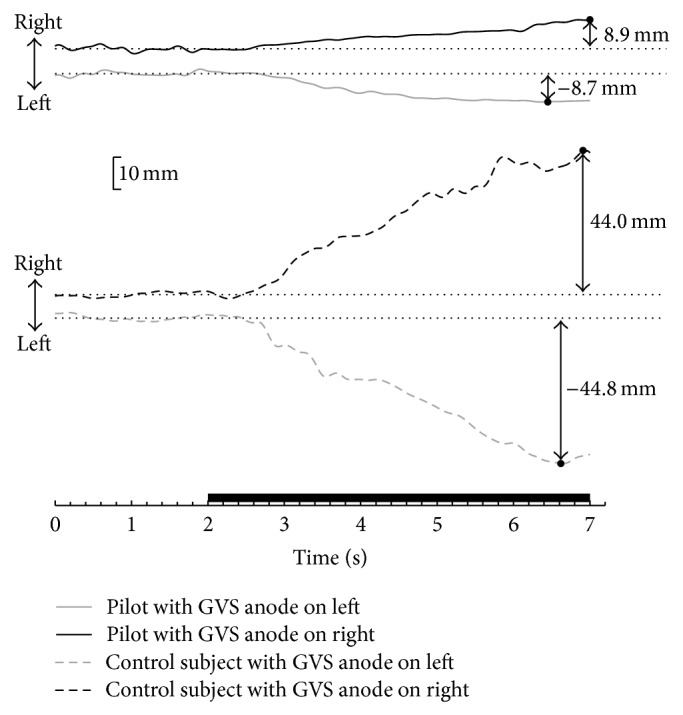
Center of pressure (COP) displacement in the medial-lateral direction of one pilot (solid line) and one control subject (dashed line) before and during a 5 s pulse of 1.0 mA GVS. The thick line on the *x*-axis indicates when GVS was applied. The dotted line stands for the average COP position during the prestimulus period, and the black dot stands for the peak COP position during the stimulus period.

Spontaneous body sway in the medial-lateral direction was slight in the absence of GVS for all subjects (Figure 1). RMS_*x*_ for control subjects was 0.8 ± 0.4 mm, which was similar to previous study [36]. RMS_*x*_ for pilots was 0.6 ± 0.2 mm. There was no significant difference between pilots and controls [*F*(1,22) = 1.141, *P* = 0.297].

### 3.3. GVS-Induced Body Sway

When 1.0 mA constant-current GVS was applied, all subjects swayed towards the anode side in the medial-lateral direction (Figure 1). In addition, the body would deviate continuously to the anode side of GVS in general. The peak displacement of COP was negative when the anode was on the left and positive when the anode was on the right.  Table 2 shows the averaged peak displacement of COP in the medial-lateral direction for pilots and controls during GVS-induced body sway according to different GVS conditions.

Statistical analysis showed a significant difference between pilots and controls for the magnitude of GVS-induced body deviation [*F*(1,44) = 31.401, *P* < 0.001], but there was no significant difference between different GVS polarities [*F*(1,44) = 0.079, *P* = 0.780].

## 4. Discussion

The results of the present study revealed that the GVS thresholds and spontaneous body sway were similar between pilots and the general populace, but the deviation in COP induced by 1.0 mA GVS was significantly different.

In this study, GVS thresholds and body sway before and during 1.0 mA bilateral bipolar GVS (larger than GVS thresholds) were tested and analyzed in order to compare the postural responses to GVS between pilots and the general populace. GVS threshold reflected the smallest stimulation that would induce postural responses, and greater values of GVS threshold meant that a larger GVS current was needed to induce body sway towards the anode side. The need for a larger current may be due to the lower sensitivity of the vestibular system to GVS. The amplitude of spontaneous body sway correlated with body stability, of which a greater value was associated with greater instability [36]. The absolute values of peak displacement during GVS-induced body sway indicated the magnitude of the peak postural responses to GVS, of which greater values stood for larger body deviation. And the larger body deviation may be due to a weaker ability to suppress vestibular illusions induced by GVS.

Balter et al. discovered that postural responses to GVS reduced after the first GVS stimulus and the reduced response could be maintained for at least 2 weeks, meaning that people could be habituated to GVS within minutes and maintain this habituation over an extended period of time [25, 26, 37]. In our GVS-induced body sway test, a trial with GVS was performed as a preliminary experiment before data collection for all subjects. Based on the results of the peak displacement of COP during GVS-induced body sway, there was no significant difference among the 3 trials. Moreover, this pretest trial could also help familiarize subjects with the sensation of GVS and the testing process.

GVS thresholds of control subjects in our study ranged from 0.2 to 0.6 mA, which were similar to previous studies [22, 32]. Additionally, GVS thresholds of pilots ranged from 0.1 to 0.6 mA, showing no significant difference. The similar GVS thresholds between the two groups indicated that the smallest stimulations to induce the postural responses were similar. It may indicate that the sensitivity of the vestibular system to GVS is not different between pilots and the general populace.

All subjects had slight spontaneous body sway during the prestimulus period. Additionally, the amplitude of spontaneous body sway was almost the same for pilots and control subjects before the onset of GVS with the anode on the right side or the left side. This meant that the stability of standing balance during the prestimulus period was similar between the two groups in all tests.

Since GVS thresholds were lower than 1.0 mA for all subjects in this study, it can be concluded that 1.0 mA GVS selected in our spontaneous and GVS-induced body sway tests was indeed a suprathreshold level for both test groups. After the 1.0 mA GVS stimulus, all subjects swayed towards the GVS anode side in the medial-lateral direction, which was in agreement with the conclusions of other studies concerning the effects of GVS on postural responses [7, 20, 21]. However, the magnitude of GVS-induced body deviation was significantly different between the two test groups. Pilots showing less GVS-induced body deviation may be benefiting from three aspects: lower spontaneous body sway, different sensitivity of vestibular system to GVS, and different response magnitude to GVS. The first two reasons may be excluded according to similar amplitude of spontaneous body sway and similar GVS thresholds between the two groups. The significantly different magnitude of GVS-induced body deviation supports the last hypothesis that pilots respond differently to GVS.

Pilots selected for this study were fully qualified, received systematic flight training, and had over 320 hours of flying experience. Systematic flight training included long-term vestibular habituation training. The purpose of the vestibular habituation training was to let pilots bear greater vestibular stimulation by improving their ability to suppress vestibular illusions, rather than the training for gymnasts to improve their balance control [25]. Therefore, less GVS-induced body deviation may be due to an improved ability to suppress vestibular illusions. For example, pilots may be aware of these vestibular illusions induced by GVS and reduce their dependence on vestibular signals, in turn reducing body sway. In the future, we will compare the postural response to GVS between trainee pilots and qualified pilots to confirm this hypothesis.

This study showed that the magnitude of the postural responses to GVS was significantly different between pilots and the general populace. Future work will study whether the postural responses to GVS can indicate a susceptibility to SD and motion sickness and thus be used to select pilots.

## 5. Conclusions

The present study verified that the magnitude of GVS-induced body deviation in pilots was significantly less than that in the general populace. Pilots showing less GVS-induced body deviation may be more capable of suppressing vestibular illusions.

## Figures and Tables

**Table 1:** GVS thresholds (mA) of pilots and control subjects during quiet stance.

GVS polarities	Pilots	Control subjects
Anode on left	0.33 ± 0.17	0.32 ± 0.12
Anode on right	0.33 ± 0.15	0.32 ± 0.11

**Table 2:** The peak displacement of COP (mm) in the medial-lateral direction for pilots and control subjects during GVS-induced body sway.

GVS polarities	Pilots	Control subjects
Anode on left	−7.2 ± 5.0	−44.9 ± 32.1
Anode on right	7.2 ± 3.8	41.6 ± 30.7

Left deviation from the initial position is expressed as negative (−) and right deviation is expressed as positive (+) in the medial-lateral direction.
